# Critical Communication: A Cross-sectional Study of Signout at the Prehospital and Hospital Interface

**DOI:** 10.7759/cureus.7114

**Published:** 2020-02-27

**Authors:** Srinivasa R Janagama, Matthew Strehlow, Aruna Gimkala, G.V. Ramana Rao, Loretta Matheson, Swaminatha Mahadevan, Jennifer A Newberry

**Affiliations:** 1 Emergency Medicine, Stanford University School of Medicine, Palo Alto, USA; 2 Research, Gunupati Venkata Krishnareddy Emergency Management and Research Institute, Hyderabad, IND; 3 Emergency Medicine Learning Centre & Research, Gunupati Venkata Krishnareddy Emergency Management and Research Institute, Hyderabad, IND

**Keywords:** communication, pre-hospital care, hand-off, healthcare quality improvement, signout, emergency medical services, pre-hospital signout

## Abstract

Introduction

Miscommunication during patient handoff contributes to an estimated 80% of serious medical errors and, consequently, plays a key role in the estimated five million excess deaths annually from poor quality of care in low- and middle-income countries (LMICs).

Objective

The objective of this study was to assess signout communication during patient handoffs between prehospital personnel and hospital staff.

Methods

This is a cross-sectional study, with a convenience sample of 931 interfacility transfers for pregnant women across four states from November 7 to December 13, 2016. A complete signout, as defined for this study, contains all necessary signout elements for patient care exchanged verbally or in written form between an emergency medical technician (EMT) and a physician or nurse.

Results

Enrollment of 786 cases from 931 interfacility transfers resulted in 1572 opportunities for signout. EMTs and a physician or nurse signed out in 1549 cases (98.5%). Signout contained all elements in 135 cases (8.6%). The mean percentage of signout elements included was 45.2% (95% CI, 43.9-46.6). Physician involvement was correlated with a higher mean percent (63.4% [95% CI, 62-64.8]) compared to nurse involvement (23.6% [95% CI, 22.5-24.8]). With respect to the frequency of signout communication, 63.1% of EMTs reported often or always giving signout, and 60.5% reported often or always giving signout; they reported feeling moderately to very comfortable with signout (73.7%) and 34.1% requested further training.

Conclusions

Physicians, nurses, and the EMTs conducted signout 99% of the time but often fell short of including all elements required for optimal patient care. Interventions aimed at improving the quality of patient care must include strengthening signout communication.

## Introduction

There are an estimated 5 million excess deaths annually in developing countries due to poor quality of care [1]. Handover of patient care is a high-risk moment and a major source of communication errors that result in adverse events [2-3]. Medical errors were projected to be the third leading cause of death following heart disease and cancer [4]. An estimated 80% of serious medical errors involve miscommunication during patient transfers [5]. Furthermore, inefficiencies in communication contribute to an estimated annual loss of $12 billion in the United States alone [6].

The World Health Organization (WHO), National Health Services (NHS), Joint Commission, and the Agency for Healthcare Research and Quality (AHRQ) have emphasized the importance of communication. They have laid out specific guidelines for safe handovers [7,8]. These guidelines differentiate the process of handoff, the transfer of responsibility for patient care, from signout, the exchange of information [9].

To date, most research on the impact of inadequate signout communication has been in-hospital studies [2]. Yet, signout communication is equally important in the prehospital setting where multiple factors influence patient signout. Signout improvement requires a multidisciplinary approach viewing the processes as social, contextual, and organizational-behavior issues [10-13]. A common language and understanding between prehospital and hospital care providers is essential to successful communication and quality patient care [12,14]. Prehospital literature on signout in low- and middle-income countries (LMICs) is scant, and their prehospital emergency service systems are still in an evolving phase. A large centralized emergency service system from India, which can serve as an archetype for other LMICs, was launched in 2005 and handled over 55 million emergencies between 2005 and 2017 [15]. Consequently, emergency medical technicians (EMTs) responding to these emergencies are performing thousands of critical handoffs and signouts daily.

A study of the signout process in an LMIC between prehospital and hospital healthcare providers is imperative given its importance to quality patient care. This study aims to assess the quality of signout during the patient handoff between prehospital and in-hospital providers.

## Materials and methods

Overview

This is a cross-sectional study of a convenience sample of pregnancy-related interfacility transfers (IFTs) by an Indian emergency medical services (EMS) provider between November 7th, 2016 and December 13th, 2016 (Monday to Saturday 8 AM to 5 PM for five weeks) across four states: Assam, Gujarat, Himachal Pradesh, and Karnataka. Research assistants’ availability defined data collection hours. IFTs were the focus of the study as they require EMTs to both receive and give signout, resulting in two data points per call. Further, the sample was confined to a single chief complaint because a) it required a uniform set of signout elements and b) calls for pregnancy-related transfers are a major focus for EMS agencies in LMICs - pregnancy-related transfers comprise over 30% of total transfers by the Indian EMS agency studied with over 30% of these being IFTs [16].

Setting

The Indian EMS agency studied, Gunupati Venkata Krishnareddy Emergency Management and Research Institute (GVK EMRI), coordinates EMS as part of a public-private partnership (PPP) with state governments. In this PPP model, the state government shares the responsibility to fund ambulance procurement; the ambulance operations; and the human resource cost of the employees working in call-center and on the ambulance. GVK EMRI provides technology and leadership to run services. Patients receive free transport from the scene to the hospital.

GVK EMRI operates centralized dispatch centers, trains EMTs to provide patient care, handles prehospital patient transfers, and implements quality improvement activities. The operations are centralized at the state level, and each state has its own operating unit. GVK EMRI transfers approximately 22,000 patients a day across all 15 states [15].

Interfacility transfers typically occur to transfer a patient from a lower level facility to a higher level facility that is better equipped to meet the patient’s medical needs. IFTs occur across all facility levels: Primary Health Centers at the primary care level; Community Health Centers, Sub-District Hospitals, and District Hospitals at the secondary care level; and Medical Colleges at the tertiary care level. GVK EMRI does the interfacility transfer upon request received from a referring facility. The referring and the receiving facility, most often, do not have a fully equipped emergency department, but rather a unit called a casualty department with a triage area, a resuscitation area, and a few observation beds; handled by a nurse and medical officers.

All EMTs undergo a 52-day training program. Initially, GVK EMRI designed this program based on the National Highway Traffic Safety Administration (United States) curriculum for EMT-Basic and tailored it to the Indian setting by including additional modules on pregnancies. Later, the program was adapted to align with recommendations the Government of India issued through the National Institute of Health and Family Welfare (NIHFW). Regardless of state, EMTs are taught to follow standard operating procedures set by the national headquarters.

Data collection

Trained research assistants (RA) identified pregnancy-related IFTs in real time using the dispatch center caller database. Each state had one to two RAs. They contacted the EMT handling the case to notify them about potential enrollment and schedule a call after the transfer was complete. Data was recorded online, using a standardized form on a secure data capture application called REDCap [17]. The form was in English, and the same form was used across all the states. We excluded cases if: the patient was transported from the field as opposed from a healthcare facility; the patient was in their first or second trimester of pregnancy; there was an ambulance dispatch issue that included dispatch cancellations, double dispatches, incorrect dispatch data; or the research assistant could not reach EMT. Finally, missing data regarding any of the exclusion criteria resulted in the exclusion of the case.

Data recorded included the healthcare provider giving and taking signout (nurse, physician or other); the method used for signout (verbal, written or both); and the elements contained in signout (Table 1). EMTs provided self-report on how often they gave and received signout, how comfortable they were with signout, and their perceptions on their need for additional training. The self-report was on a 5-point Likert scale.

**Table 1:** Complete signout definition

	At referring facility	At receiving facility
Person	Given by nursing staff or physician	Received by nursing staff or physician
Method	Either verbal or written	Either verbal or written
Signout elements	1. Primary diagnosis	1. Primary diagnosis
	2. Interventions at the referral facility	2. Interventions at the referral facility
	3. Length of stay at the referral facility	3. Length of stay at the referral facility
	4. Obstetric history	4. Obstetric history
	5. Past medical history	5. Past medical history
	6. Usual medications	6. Usual medications
	7. Allergies	7. Allergies
	8. Reason for transfer	8. Vital signs during transport
		9. Interventions during transport

Participants provided verbal consent for care, transport, and follow-up at the time of transport as per the GVK EMRI standard operating procedures. The study was reviewed and approved by the Stanford Institutional Review Board (IRB) (IRB 36066) as well as the GVK EMRI ethics committee.

Definition of complete signout

A complete signout, as defined for this study, contains all necessary signout elements for patient care exchanged verbally or in written form between EMT and a physician or nurse (Table 1). Standardized signout methods group signout information into categories. Examples of such standardized formats include the SBAR and the I-PASS. SBAR includes situation, background, assessment, and recommendations [18]. I-PASS includes illness severity, patient summary, action list, situation awareness and contingency planning, and synthesis by the receiver [19]. Signout elements included in our definition of a complete signout represented the same information conveyed by the aforementioned SBAR and I-PASS categories.

Data analysis

The primary outcome was the frequency of complete signout as defined above. The secondary outcomes were 1) the proportion of required elements included in a given signout, and 2) the EMT’s self-reported competence with signout. Frequencies, means, and 95% confidence intervals were included for reported descriptive statistics. The frequency of missing data was shown within the tables. Data analysis was with SAS Enterprise Guide, v.7.15 (SAS Institute Inc., Cary, USA) and R v.3.5.1 with R- studio v.1.1.447, RStudio, Inc.

## Results

Overview

Research assistants selected 931 cases for data collection. A total of 786 (84.4%) cases were enrolled after excluding 145 (15.6%) that met exclusion criteria. Data related to the three exclusion criteria were missing in 40 (4.3%) cases, and these cases were excluded. In the remaining 105 (11.3%) cases, 32 (3.4%) cases were field transfers and not IFTs, 45 (4.8%) cases had patient either in the first or second trimester of pregnancy, and 37 (3.9%) cases had a dispatch issue. Some cases met one or more of the three exclusion criteria.

Demographic characteristics

Among the 786 cases enrolled, Assam contributed 200 (25.5%), Gujarat 152 (19.3%), Himachal Pradesh 111 (14.1%), and Karnataka 323 (41.1%) cases. A basic life support ambulance was dispatched for 661 (84.1%) of the 786 transfers. A basic EMT handled 99% (777) of transfers. Overall, 586 (74.6%) EMTs had obstetric refresher training. The referrals were predominantly from primary health centers (267 [34%]), community health centers (283 [36%]), and sub-divisional hospitals (137 [17.4%]). District hospitals received a majority of the cases (357 [45.4%]). The medical college hospitals and sub-divisional hospitals received a major part of the rest (178 [22.6%] and 116 [14.8%], respectively).

Signout characteristics - complete signout

From 786 IFTs, there were 1572 occasions of signout as signout occurs both at the referring and the receiving facility. The EMT and hospital personnel interacted either verbally or in written form or both on 1549 (98.5%) occasions. All required elements were included in signout on 135 (8.6%) occasions. A nurse or a physician at the referring facility gave a complete signout to the EMT on 69 (8.8%) occasions and the EMT gave a complete signout to a nurse or a physician at the receiving facility at a similar frequency (64 [8.1%]). Overall, signout was complete on 133 (8.4%) occasions - on two occasions all elements were included but signout was not performed by a physician or nurse. Least frequently included elements were length of stay in referring facility (269 [17.1%]), interventions during transport (171 [21.8%]) and referring facility interventions (374 [23.8%]) (Table 2).

**Table 2:** Signout characteristics *Reason for transfer was not part of signout elements documented at the receiving facility. Vital signs during transport and Interventions during transport were not part of signout elements included at the referring facility.

	Referring facility		Receiving facility	Overall
	n=786	n=786		n=1572
Complete signout				
n (%)	69 (8.8)	64 (8.1)		133 (8.4)
Signout content included				
Mean percent	42.8	47.7		45.2
95% CI	40.8 - 44.8	45.8 - 49.5		43.9 - 46.6
Signout elements included	n (%)	n (%)		n (%)
All elements	69 (8.8)	66 (8.4)		135 (8.6)
Primary diagnosis	448 (57.0)	443 (56.4)		891 (56.7)
Reason for transfer*	448 (57.0)	-		448 (57.0)
Interventions at referral facility	127 (16.2)	247 (31.4)		374 (23.8)
Length of stay at referral facility	125 (15.9)	144 (18.3)		269 (17.1)
Obstetric history	496 (63.1)	461 (58.7)		957 (60.9)
Past medical history	442 (56.2)	465 (59.2)		907 (57.7)
Usual medications	295 (37.5)	318 (40.5)		613 (39.0)
Allergies	304 (38.7)	320 (40.7)		624 (39.7)
Vital signs during transport*	-	671 (85.4)		671 (85.4)
Interventions during transport*	-	171 (21.8)		171 (21.8)
No elements	6 (0.8)	6 (0.8)		12 (0.8)
Missing	2 (0.3)	4 (0.5)		6 (0.4)
Signout person				
Physician	435 (55.3)	412 (52.4)		847 (53.8)
Nursing staff	336 (42.7)	366 (46.6)		702 (44.7)
Others	13 (1.7)	4 (0.5)		17 (1.1)
No signout	1 (0.1)	0 (0.0)		1 (0.1)
Missing	1 (0.1)	4 (0.5)		5 (0.3)
Signout method				
Both verbal and written	622 (79.1)	631 (80.3)		1253 (79.7)
Verbal	16 (2)	8 (1)		24 (1.5)
Written	140 (17.8)	130 (16.5)		270 (17.1)
No signout	1 (0.1)	0 (0)		1 (0)
Missing	7 (0.9)	17 (2.2)		24 (1.6)

Signout characteristics - state variations

States differ in the way they gave signout. In Karnataka, the nursing staff was involved in signout most often (635 [98.3%]), whereas, in Himachal Pradesh, it was physicians (213 (95.9%)). In Himachal Pradesh, personnel predominantly exchanged written forms (220 [99.1%]) without verbal interaction. The included signout elements varied between states and inclusion of a particular element varied across the states. Providers in Karnataka most often included obstetric history (601 [93%]), whereas those in Assam and Gujarat regularly included primary diagnosis (379 [94.8%] and 295 [97%]) and past medical history (341 [85.2%] and 243 [79.9%]) (Table 3). This pattern is suggestive of localized, state-specific processes and culture around signout.

**Table 3:** Signout characteristics - state variations *The denominator for “reason for transfer” included referring facility signouts only. The denominator for “vital signs during transport” and “interventions during transport” included receiving facility signouts only. They were as follows: Assam; 200; Gujarat: 152; Himachal Pradesh: 111; and Karnataka: 323.

	Assam	Gujarat	Himachal Pradesh	Karnataka
	n=400	n=304	n=222	n=646
	n (%)	n (%)	n (%)	n (%)
Signout elements				
Primary diagnosis	379 (94.8)	295 (97.0)	210 (94.6)	7 (1.1)
Reason for transfer*	182 (90.0)	143 (94.0)	106 (95.4)	17 (5.2)
Interventions at the referral facility	4 (1.0)	114 (37.5)	209 (94.1)	47 (7.3)
Length of stay at the referral facility	2 (0.5)	65 (21.4)	201 (90.5)	1 (0.2)
Obstetric history	45 (11.2)	107 (35.2)	204 (91.9)	601 (93.0)
Past medical history	341 (85.2)	243 (79.9)	200 (90.1)	123 (19.0)
Usual medications	280 (70.0)	156 (51.3)	169 (76.1)	8 (1.2)
Allergies	332 (83.0)	146 (48.0)	145 (65.3)	1 (0.2)
Vital signs during transport*	172 (86.0)	137 (90.2)	78 (70.2)	284 (88.0)
Interventions during transport*	1 (0.4)	91 (68.8)	75 (67.6)	4 (1.2)
Signout person				
Physician	376 (94)	255 (83.9)	213 (95.9)	3 (0.5)
Nursing staff	16 (4)	46 (15.1)	5 (2.3)	635 (98.3)
Signout method				
Both verbal and written	345 (86.2)	292 (96.1)	0 (0)	616 (95.4)
Verbal	9 (2.2)	4 (1.3)	1 (0.5)	10 (1.5)
Written	32 (8)	2 (0.7)	220 (99.1)	16 (2.5)

Signout characteristics - signout content

Overall, inclusive of referring and receiving facilities in all states, facility physician involvement resulted in the inclusion of more signout elements on an average (63.4% [95% CI 62.0-64.8]) compared to nursing staff involvement (23.7% [95% CI 22.5-24.8]) (Wilcoxon rank-sum, p<0.001). Referring facility signout on an average had 42.8% (95% CI 40.9-44.8) of elements compared to 47.7% (95% CI 45.8-49.5) at receiving facilities (Table 4). Among the states studied, Karnataka included the least number of signout elements on average (20.4%, 95% CI [19.7-21.1]) and Himachal Pradesh included the most on average (84.9%, 95% CI [81.9-87.9]). Of note, it was physicians that most often were involved in signout in Himachal Pradesh, whereas in Karnataka it was nursing staff (Table 3).

**Table 4:** Mean percentage of signout elements included in signout *From a total of 786 signouts at referring facility, signout element data was missing in two cases, and there was no signout in one case. ^!^Providers other than physician or nursing staff gave signout in 13 cases. #From a total of 786 signouts at receiving facility, signout element data was missing in four cases. ^^^Provider data was missing in spite of having a signout in two cases, and a provider other than physician or nursing staff gave signout in four cases.

		Referring facility		Receiving facility	
State	Provider type	Signouts	Signout content	Signouts	Signout content
		n	Mean % (95% CI)	n	Mean % (95% CI)
All states	All providers	783^*^	42.8 (40.9-44.8)	782^#^	47.7 (45.8-49.5)
All states	Physician	435^!^	63.4 (61.5-65.4)	410^^^	63.3 (61.3-65.4)
	Nursing staff	335^!^	16.8 (15.7-17.8)	366^^^	30.0 (28.2-31.7)
Assam	Physician	186	57.3 (55.6-59.1)	189	51.0 (49.1-52.9)
	Nursing staff	9	20.8 (06.4-35.2)	6	33.3 (00.0-72.4)
Gujarat	Physician	140	54.1 (51.8-56.5)	115	65.1 (62.4-67.9)
	Nursing staff	10	53.8 (46.4-61.1)	36	66.0 (60.6-71.5)
Himachal Pradesh	Physician	109	85.9 (81.6-90.1)	103	84.6 (80.2-88.9)
	Nursing staff	0	-	5	73.3 (33.6-100)
Karnataka	Physician	0	-	3	40.7 (24.8-56.7)
	Nursing staff	316	15.5 (14.8-16.2)	319	25.1 (24.2-26.1)
Assam	All providers	198	55.1 (53.0-57.2)	198	50.3 (48.2-52.4)
Gujarat		151	53.9 (51.7-56.1)	151	65.3 (62.9-67.8)
Himachal Pradesh		111	85.5 (81.2-89.7)	110	84.3 (80.1-88.6)
Karnataka		323	15.5 (14.8-16.2)	323	25.3 (24.3-26.2)

EMT perspective on the signout process

Overall, EMTs felt moderately to very comfortable giving signout (574 [73.7%]). The majority of EMTs reported giving (494 [63.1%]) and receiving (474 [60.5%]) signout "most of the time" or "always”. A minority of EMTs, 267 (34.1%), felt they require further training in signout practices. However, EMTs in Assam differ in their opinions compared to other states. Most report feeling only slightly comfortable giving signout (174 [87.9%]), and they give and receive signout only some of the time (179 [90.4%] and 178 [89.4%]). Moreover, Assam EMTs near-universally felt they require further training (192 [96.5%]) (Table 5).

**Table 5:** EMT self-report EMT, emergency medical technician; IFT, interfacility transfer.

	Assam	Gujarat	Himachal Pradesh	Karnataka	Overall
	n (%)	n (%)	n (%)	n (%)	n (%)
How comfortable do you feel giving sign out? (n=779)					
Not at all comfortable	1 (0.5)	0 (0)	1 (0.9)	0 (0)	2 (0.3)
Slightly comfortable	174 (87.9)	2 (1.4)	1 (0.9)	0 (0.0)	177 (22.7)
Somewhat comfortable	10 (5.1)	3 (2.0)	2 (1.8)	11 (3.4)	26 (3.3)
Moderately comfortable	5 (2.5)	1 (0.7)	105 (94.6)	306 (94.7)	417 (53.5)
Very comfortable	8 (4.0)	141 (95.9)	2 (1.8)	6 (1.9)	157 (20.2)
How often do you give sign out to a health care provider when dropping off a patient to a receiving facility after a field call or an IFT? (n=783)					
Never	1 (0.5)	0 (0)	4 (3.6)	0 (0)	5 (0.6)
Rarely	7 (3.5)	0 (0)	1 (0.9)	9 (2.8)	17 (2.2)
Some of the time	179 (90.4)	20 (13.2)	5 (4.5)	63 (19.5)	267 (34.1)
Most of the time	10 (5.1)	21 (13.9)	101 (91.0)	251 (77.7)	383 (48.9)
Always	1 (0.5)	110 (72.8)	0 (0)	0 (0)	111 (14.2)
How often do you receive sign out from a health care provider when picking up a patient for an IFT? (n=783)					
Never	0 (0)	0 (0)	2 (1.8)	2 (0.6)	4 (0.5)
Rarely	8 (4.0)	0 (0)	1 (0.9)	12 (3.7)	21 (2.7)
Some of the time	178 (89.4)	21 (13.9)	7 (6.4)	78 (24.1)	284 (36.3)
Most of the time	13 (6.5)	20 (13.2)	100 (90.9)	231 (71.5)	364 (46.5)
Always	0 (0)	110 (72.8)	0 (0)	0 (0)	110 (14.0)
Would you like more training on how to give sign out and how to receive sign out? (n=782)					
Not required	7 (3.5)	132 (88.6)	101 (91.0)	202 (62.5)	442 (56.5)
Not sure	0 (0)	2 (1.3)	0 (0)	71 (22.0)	73 (9.3)
Required	192 (96.5)	15 (10.1)	10 (9.0)	50 (15.5)	267 (34.1)

## Discussion

This first of its kind study of prehospital - hospital signout in an LMIC revealed that healthcare providers conducted signout in 99% of the cases. There were variations across states concerning the type of provider involved in signout, and the content varies depending on the provider type. The content of signout was more comprehensive when a physician was involved in signout with the EMT. The mean percent of signout elements included (45.2% [95% CI 43.9-46.6]) was comparable to earlier studies performed in countries with mature emergency care systems. In a study by Carter *et al*. on information loss during signout of trauma patients by emergency medical services, the EMTs and paramedics communicated on average 4.9 (95% CI 4.55-5.24) signout elements from a total of 16 [20]. In a study by Goldberg *et al*. on the content of EMS signout, 78% (95% CI 70.0-86.7) of signouts included the chief complaint, and 57% (95% CI 46.7-66.7) of signouts included vital signs [21].

Reviews on prehospital signout have identified multiple factors that influence signout. Busy emergency departments (ED) with overloaded staff forced to handle multiple tasks at the same time in a chaotic environment offer a greater potential for multiple interruptions and passive listening during handoff [10,14,22]. Interruptions lead to multiple attempts to handover, paving way for frustration and information loss [14]. Moreover, a lack of structured signout compounded by a lack of common language and understanding between the ambulance staff and hospital personnel and a lack of assigned personnel to handover may further complicate the transfer [14,22]. Further, it is reported that only 75% of information gets into patient notes and only 50% of ED staff take ambulance charts into consideration [23-24]. Any of these factors may contribute to the lower thoroughness of signout observed in this study. An editorial article on patient transfers in LMICs noted such transfers to be dependent on multiple factors and emphasized the influence of the cultural divide between the ambulance and the hospital care providers [25].

From among these factors, structured signout is afforded much importance, and multiple signout tools have been developed and studied. Despite not having a formal process or utilizing a specific signout tool, at the state level an innate structure has developed to support the signout process as evidenced by the distinct interstate variation identified in this study. Further, states are fairly consistent with the type of provider involved in signout, the method used, and the number and type of signout elements included. Use of checklists; standardization of signout components with the use of mnemonics; and computerization of such checklists have been shown to improve signout efficiency in other settings and may improve the quality of signout in LMICs as well [2,26-27]. Unfortunately, there is no clearly established best practice when it comes to the use of signout mnemonics. Riesenberg *et al*. in their 2008 systematic review identified 24 signout mnemonics used in and out of hospital settings [28]. Despite the lack of established best practice, when implemented, mnemonics and standardized signouts may improve quality. Starmer *et al*. in their study on changes in medical errors after implementation of the I-PASS system noted a 23% decrease in the rate of medical-errors (24.5 vs. 18.8 per 100 admissions, p<0.001) and a 30% decrease in the rate of preventable adverse events (4.7 vs. 3.3 events per 100 admissions, p<0.001) [29].

Standardization with mnemonics alone may be insufficient for improving communication and the quality of emergency care in India and other countries. Reviews on prehospital signout suggest shared mental models that achieve a common language and understanding between prehospital and hospital care providers as important for strengthening communication [11-12]. A shared mental model consists of: (1) understanding of technology or the equipment being used; (2) knowledge about the way to accomplish a task within the environment; (3) perceptions of each team member’s role and how the team members will collaborate to communicate the information; and (4) shared understanding of each team member’s knowledge, skills, attitudes, strengths, and limitations [30]. The ambulance and hospital interface is characterized by personnel with distinct levels of education and training coming from different organizational cultures. This environment can be chaotic because the providers are often multitasking. Moreover, the situation is compounded by tighter time limits and incompatible health record systems. Given these unique characteristics of the ambulance-hospital interface, a shared mental model seems essential.

Of note, the EMT-B training program has a session on communication across the states included in the study. Furthermore, based on a review of the literature and direct observation, they are also developing a novel continuing education module to promote consistent signout communication. In the future, integrated sessions for EMTs and hospital care providers in LMICs, and potentially elsewhere, should cultivate the development of a shared mental model and team-based learning.

Limitations

This study provides a snapshot of signout practices but does not identify the cause for the low frequency of a complete signout. Further, the signout elements included were identified at a broad level and lacked specific details. This study was based on EMT’s self-report as a screening of the written documents and direct observation of the handover was not possible at the time of this study. Moreover, another potential limitation would be the Hawthorne effect. EMTs were aware that data collection would occur which may influence the reporting of signout elements. We believe the recall bias is limited as the EMT was called immediately after the patient was transferred. Finally, the reason for the transfer was included in referring facility signout and missed in receiving facility signout during data collection.

## Conclusions

Physicians or the nurses and the EMT communicated on 99% of signout opportunities and included 45.2% of recommended signout content. The rate of a complete signout by EMTs was comparable to the rate of a complete signout by physicians and nurses. Improvement requires a multifactorial interdisciplinary approach - one that accounts for the unique ambulance hospital interface. Recognition by ambulance and hospital-based providers on the critical nature of signout in providing quality care is imperative. Interventions should include a standardized signout process, coordinated training that fosters a shared mental model, and integrated information systems.
